# Commentary: Exploring the clinical features of minimally verbal autistic children

**DOI:** 10.3389/fpsyt.2026.1892115

**Published:** 2026-07-08

**Authors:** Ashley Priscilla Good

**Affiliations:** 2m Foundation, Hillsborough, CA, United States

**Keywords:** ADOS-2, autism assessment, expressive language, minimally verbal autism, motor autism, motor impairment, motor phenotyping, speech-motor control

## Introduction

Guerrera et al. (1) address an important and underserved population: minimally verbal autistic children. Their effort to characterize heterogeneity in cognitive and language profiles is valuable. However, several methodological assumptions constrain the interpretation of their findings, particularly where limited speech is treated as a proxy for broader cognitive or developmental severity. This concern is especially salient because the ADI-R and ADOS were not developed for individuals with severe vision, hearing, and/or motor impairments (2). In minimally verbal autism, where motor planning, speech initiation, response timing, and sensory regulation may be impaired, observed speech output cannot be assumed to reflect underlying language capacity or cognition.

## ADOS classification and circular inference

A central concern is the use of ADOS-2 Module 1 assignment and item scores to define minimally verbal status and then anchor downstream comparisons of cognition and development. ADOS was developed as a standardized behavioral observation tool to support diagnostic classification, not as a direct measure of expressive language capacity or underlying cognition. When speech output contributes to module assignment and severity scores, and those same classifications are then interpreted as evidence of developmental limitation, circularity is introduced.

This is not a minor technical issue. Torres et al. (3) show that the research ADOS contains nonlinear scoring dynamics and hidden priors that can distort group comparisons, particularly when motor and language profiles differ. Children with intact nonverbal cognition but constrained speech production may therefore be misclassified as more globally impaired by an instrument not designed to separate language capacity from motor execution.

ADOS also operates beyond research as a clinical gatekeeper for diagnosis, service access, and intervention pathways. In the United States, autism diagnosis and associated evaluation practices often influence access to publicly and privately funded services, including ABA-based interventions. State insurance mandates require or support coverage for autism-related services across the United States and have reshaped the autism service workforce, including growth in board-certified behavior analysts (4, 5). This context matters because ADOS-derived classifications may influence not only research conclusions but also service pathways and reimbursement decisions. Broderick’s analysis of the “autism industrial complex” further underscores how autism assessment and intervention have become embedded in broader institutional and commercial systems (6). This is consequential because ADOS can influence clinician diagnostic certainty and is often deployed when diagnostic confidence is lower (7).

## Speech output is not cognitive capacity

Guerrera et al.’s own data caution against equating limited speech with diminished cognitive capacity: a subset of minimally verbal participants demonstrated nonverbal IQ scores within or near the average range. This dissociation is consistent with prior work showing that expressive speech depends on motor planning, coordination, and timing, and that impairments in these systems can severely constrain verbal output without implying reduced understanding or intent (8–10).

Language severity is therefore unlikely to map onto a single domain. It may reflect interacting motor, sensory, and autism-related constraints: orofacial praxis, postural control, sensory modulation, attention, initiation, and the social demands of rapid reciprocal exchange. Even nominally “nonverbal” cognitive testing remains situated within a behavioral frame that can privilege speed, compliance, and motor execution. Reduced speech should therefore be interpreted as performance under constraint, not as direct evidence of diminished capacity.

## Motor constraints on communication

The most consequential limitation is the absence of motor assessment as a core explanatory domain for speech limitations. Motor impairments, including dyspraxia, atypical postural control, and motor sequencing deficits, are pervasive across autism and are associated with social and communicative differences (11–13). These systems support not only spoken language but also handwriting, typing, gesture, and other forms of communication.

Genetic etiologies also illustrate this motor-language intersection. For example, SHANK3-related Phelan-McDermid syndrome is associated with hypotonia, motor impairment, autism features, and absent or severely delayed speech (14, 15). However, such etiologies explain only a subset of minimally verbal autism; they should motivate, rather than replace, direct motor and sensory phenotyping across the broader population.

Recent work using wearable sensors, motion capture, and surface electromyography in minimally verbal autistic individuals shows increased motor noise, reduced coordination, and atypical facial muscle activation during attempted speech, suggesting constrained motor control rather than absence of communicative intent (10, 16, 17). Neuroimaging and neurophysiology studies further implicate motor and speech-motor regions in language-related outcomes (13, 18). Without direct assessment of motor systems, attributing limited speech primarily to language or cognition risks mislocating the source of difficulty.

## Discussion

We do not suggest that Guerrera et al.’s study lacks value, nor that ADOS is intrinsically inappropriate. As an assay of observed social interaction within defined constraints, ADOS can contribute to clinical characterization and research cohort description. The concern is category error: using ADOS module assignment or observational language output as a proxy for expressive language capacity or cognitive potential in minimally verbal children.

A clearer developmental direction for minimally verbal autistic children begins with separating capacity from performance and identifying treatable constraints. Future work should acknowledge ADOS/ADI-R limitations in individuals with significant motor impairments (2), known measurement bias by spoken language level and nonverbal mental age (19), and evidence implicating motor planning and execution in speech output. A motor-informed pathway should first establish whether a motor subtype is present, then use objective measures of movement, speech-motor effort, facial/orofacial control, response timing, and dyadic interaction to improve clinical characterization and guide individualized intervention planning. Such approaches would better support minimally verbal autistic individuals by identifying constraints on expression rather than mistaking those constraints for absence of understanding.
